# Acrolein contributes strongly to antimicrobial and heterocyclic amine transformation activities of reuterin

**DOI:** 10.1038/srep36246

**Published:** 2016-11-07

**Authors:** Christina Engels, Clarissa Schwab, Jianbo Zhang, Marc J. A. Stevens, Corinne Bieri, Marc-Olivier Ebert, Kristopher McNeill, Shana J. Sturla, Christophe Lacroix

**Affiliations:** 1 Laboratory of Food Biotechnology, Institute of Food, Nutrition and Health, Department of Health Sciences and Technology, ETH Zurich, Zurich, Switzerland; 2 Laboratory of Food Nutrition and Toxicology, Institute of Food, Nutrition and Health, Department of Health Sciences and Technology, ETH Zurich, Zurich, Switzerland; 3 Laboratory of Organic Chemistry, Department of Chemistry and Applied Sciences, ETH Zurich, Zurich, Switzerland; 4 Laboratory of Environmental Chemistry, Institute of Biogeochemistry and Pollutant Dynamics, Department of Environmental Systems Science, ETH Zurich, Zurich, Switzerland

## Abstract

Glycerol/diol dehydratases catalyze the conversion of glycerol to 3-hydroxypropionaldehyde (3-HPA), the basis of a multi-component system called reuterin. Reuterin has antimicrobial properties and undergoes chemical conjugation with dietary heterocyclic amines (HCAs). In aqueous solution reuterin is in dynamic equilibrium with the toxicant acrolein. It was the aim of this study to investigate the extent of acrolein formation at various physiological conditions and to determine its role in biological and chemical activities. The application of a combined novel analytical approach including IC-PAD, LC-MS and NMR together with specific acrolein scavengers suggested for the first time that acrolein, and not 3-HPA, is the active compound responsible for HCA conjugation and antimicrobial activity attributed to reuterin. As formation of the HCA conjugate was observed *in vivo,* our results imply that acrolein is formed in the human gut with implications on detoxification of HCAs. We propose to re-define the term reuterin to include acrolein.

Biological and chemical functional activities are a key to processes that dictate the environmental and health impact of microbial ecosystems. For example, intestinal microbiota modifies primary bile acids released from the host to secondary bile acids with potential links to carcinogenesis^1^. On the other hand, the gut microbiota ferments host and dietary derived carbohydrates to short chain fatty acids, which are involved in the regulation host-microbiota interactions and might suppress cancer development or inflammation^1^. Glycerol is an abundant carbohydrate source in the gut and becomes available as a product of luminal microbial fermentations, digestion of luminal fats, sloughed mucus and desquamated epithelial cells, and intestinal clearing of endogenous plasma^2^. Members of the genera *Klebsiella, Enterobacter, Citrobacter, Clostridium*, and *Lactobacillus,* and *Eubacterium hallii* are capable of reductive glycerol metabolism^3^,^4^,^5^,^6^,^7^,^8^,^9^. Hereby, glycerol is converted to 3-HPA, a reaction that is catalyzed by a cobalamin-dependent glycerol/diol dehydratase^10^. In most producer strains, 3-HPA is immediately reduced to 1,3-propanediol by a 1,3-propanediol dehydrogenase during growth^11^. Certain lactobacilli, however, excrete 3-HPA in low glucose environments, and *E. halli* does not seem to further metabolize 3-HPA due to an apparent lack of 1,3-propanediol dehydrogenase activity^12^,^13^,^14^,^15^. In aqueous solution 3-HPA exists in an equilibrium with mainly its hydrate 1,1,3-propanetriol and its dimer 2-(2-hydroxyethyl)−4-hydroxy-1,3-dioxane^16^; other multimeric forms have also been reported^17^,^18^. This dynamic system has been called reuterin after *Lactobacillus reuteri,* the best studied model organism for 3-HPA production (Fig. 1).

Reuterin exhibits inhibitory activity against a broad range of Gram-positive and Gram-negative bacteria, yeasts, molds, and protozoa, including various food spoilers and pathogens, lactic acid bacteria used in food fermentations, and also microorganisms residing in the mammalian gastro-intestinal tract^5^,^19^,^20^,^21^,^22^,^23^. The mechanistic basis of reuterin’s antimicrobial activity has been proposed to be an imbalance in cellular redox status resulting from reactions of 3-HPA with free thiol groups, causing the depletion of glutathione (GSH) and modification of proteins, including functional enzymes^20^,^21^.

In addition to its well-investigated antimicrobial functions, reuterin is implicated in the conjugation of heterocyclic amines (HCAs), a process of potential relevance to the bioavailability of toxicants in the human gut^24^,^25^. Analogous to antimicrobial activity, it was hypothesized previously that HCA transformation is a result of the reaction of HCA with 3-HPA. For example, the HCA 2-amino-1-methyl-6-phenylimidazo[4,5-b]pyridine (PhIP), an amino acid pyrolysis product formed when meat is cooked at high temperatures, is transformed to its conjugate metabolite 7-hydroxy-5-methyl-3-phenyl-6,7,8,9-tetrahydropyrido[3′,2′:4,5]imidazo[1,2-α]pyrimidin-5-ium chloride (PhIP-M1) in a microbial process that requires glycerol^26^. *L. reuteri* and more recently *E. hallii* have been demonstrated to be competent in mediating this transformation^24^,^26^. Because HCAs are mutagenic and possible human carcinogens contributing to the increased risk of colorectal cancer from eating meat, a detailed understanding of their physiological transformation pathways is a critical component of risk analysis^27^,^28^,^29^,^30^.

Antibacterial activity and glycerol-dependent biotransformation of HCAs have been attributed to 3-HPA. However, as Voisenet already discovered in the early 20^th^ century^31^,^32^, 3-HPA spontaneously dehydrates to form acrolein, a cytotoxic electrophile^33^,^34^ and a genotoxic mutagen^35^. We hypothesized that the spontaneous conversion of 3-HPA to acrolein contributes to functionalities typically attributed to reuterin, namely antimicrobial activity and HCA conjugation. To our knowledge, this question has never been addressed, possibly because the highly dynamic reuterin system is challenging to analyze in a quantitative manner, even without accounting for the potential formation of acrolein. Accurate analysis of 3-HPA is difficult because no commercial standards are available for external calibration. The concentration of 3-HPA and acrolein in reuterin were previously determined using nuclear magnetic resonance (^13^C-NMR) analysis^16^, high-pressure liquid chromatography (HPLC) analysis with refractive index (RI) detection^23^,^36^, and gas chromatography with mass spectrometry detection (GC-MS) analysis after derivatization or after solid-phase microextraction^15^,^37^. The colorimetric detection method frequently used to quantify 3-HPA^38^ was developed originally for the analysis of acrolein. No established method has therefore been effective for the simultaneous quantification of 3-HPA and acrolein with sufficient sensitivity and specificity.

We aimed to quantify acrolein present in reuterin solutions at physiological conditions and evaluate its relevance for functionality typically attributed to 3-HPA, including antimicrobial activity and HCA conjugation. We developed therefore novel multiple analytical approaches for measuring all components of the reuterin system, including acrolein, in complex mixtures. We characterized the dynamics of the interconversion of acrolein and 3-HPA in aqueous buffers and in bacterial culture broth and investigated the relevance of the 3-HPA-to-acrolein transformation to the antimicrobial properties of reuterin by determining minimum inhibitory concentrations of acrolein and 3-HPA toward bacterial indicators in the presence and absence of acrolein scavengers. Furthermore, the involvement of 3-HPA to acrolein in microbe-mediated chemical conjugation of glycerol was investigated.

## Results

### Multi-method analysis of the dynamic reuterin system

#### Quantification of acrolein and 3-HPA and investigation of their interconversion in buffer

We integrated data from ion-exclusion chromatography with pulsed-amperometric detection (IC-PAD), NMR and ultra-performance liquid chromatography coupled with electrospray ionization tandem mass spectrometry in multiple reaction monitoring mode (MRM UPLC-ESI-MS/MS) to gain information regarding all compounds of the reuterin system namely 3-HPA, its hydrate 1,1,3-propanetriol, its dimer 2-(2-hydroxyethyl)-4-hydroxy-1,3-dioxane and also acrolein.

Quantitative investigation of compositional shifts between 3-HPA and acrolein were enabled using a newly developed IC-PAD method, entailing optimization of the triple-potential waveform electrode potentials. The novel method allowed for the first time concurrent detection and quantification of 3-HPA, acrolein, glycerol, and 1,3-propanediol (Fig. 2a). Potentials of the newly established waveform were E_1_ = 0.3 V (t_1_ = 0.31 s), E_2_ = 1.25 V (t_2_ = 0.34 s, t_int_ = 0.02 s), and E_3_ = −0.4 V (t_3_ = 0.39 s); the integration period was placed at the end of the oxidative step E_2_ which reduces baseline noise resulting in improved sensitivity^39^. Waveform potentials were similar to those used in other studies concerning glycerol or acrolein individually^39^,^40^. Calibration curves ranged from 0.05 to 6 mM for 3-HPA (Limit of detection (LOD) = 7.5 μM), from 0.04 to 1 mM for acrolein (LOD = 4.4 μM), from 0.75 to 20 mM for glycerol (LOD = 104.9 μM) and from 0.10 to 13 mM for 1,3-propanediol (LOD = 17.1 μM).

Formation of 3-HPA and acrolein during incubation of *L. reuteri* cells in 200 mM glycerol solutions was analyzed using the newly established IC-PAD method. Experiments confirmed production of 3-HPA from glycerol, but also revealed the presence of acrolein at 25 °C after 60 min (Fig. 2d). Amounts of both compounds continued to increase reaching final concentrations of about 0.2 mM acrolein and 64.4 mM 3-HPA after 3 h. The amount of 1,3-propanediol formed remained below 10 mM (Fig. 2d).

Pure 3-HPA was recovered by its isolation from reuterin by solid-phase extraction with silica gel 60. The absence of acrolein in the resulting solution was confirmed by IC-PAD analysis (Fig. 2e). Resulting pure 3-HPA, as well as commercially available acrolein, were used to characterize the kinetic behavior of the 3-HPA/acrolein interconversion process, which was assessed under a range of physiologically related conditions (T = 4–45 °C; pH 6–9, Table 1). 3-HPA was not detected in acrolein samples and acrolein was not detected in 3-HPA samples at pH 4 at 4 °C after 48 h, suggesting that these compounds do not interconvert at low temperature and acidic pH. Similarly, no acrolein was detectable in 3-HPA samples at pH 7 at 4 °C. However, small amounts of 3-HPA were present in acrolein samples at pH 7 at 4 °C (≤ 0.06 mM). Under the conditions investigated (T = 4–45 °C; pH 6–9) the conversion was reversible, with 3-HPA being favored (Table 1). Acrolein polymerization competed with the 3-HPA/acrolein interconversion, as observed previously^41^,^42^,^43^,^44^, therefore, decay via polymerization was accounted for in the kinetic model used to describe the dynamics of the interconversion of 3-HPA and acrolein (Table 1, Equation 1). Higher temperatures and a more alkaline pH resulted in faster 3-HPA to acrolein dehydration rates. At conditions prevailing in the human colon (37 °C, pH around 5.6–7 depending on section), the ratio of 3-HPA:acrolein was 86:14.

#### Acrolein/3-HPA interconversion in bacterial culture broth

After establishing the presence of acrolein in buffered reuterin solutions, acrolein concentrations were also determined in lysogeny broth (LB) medium used for antibacterial activity testing. Hereby, concentrations of reuterin, 3-HPA and acrolein were analyzed before and after their addition to LB medium (Fig. 3a–c). As the analysis of undiluted bacterial culture broth caused significant deterioration of the platinum electrode surface in IC-PAD experiments, the analysis was performed once for 3-HPA and acrolein and in duplicate for reuterin solutions, furthermore control samples containing defined amounts of glycerol were periodically analyzed to detect and account for changes in electrode response.

Acrolein was already present at just below 0.1 mM in reuterin solutions containing 6.9 mM HPA prior to reuterin addition to bacterial culture broth (Fig. 3a) and levels immediately decreased to 0.02 mM after mixing with LB medium at 37 °C. When pure (i.e. initially acrolein-free) 3-HPA was added at 6.0 mM to LB medium, acrolein became immediately detectable, and continued to increase until reaching a maximum at 0.05 mM after 160 min of incubation. Thereafter acrolein concentrations decreased to 0.01 mM until the end of incubation (Fig. 3b). Levels of acrolein decreased immediately from 7.1 mM to 1.5 mM when pure (i.e. initially 3-HPA free) acrolein was mixed with LB broth at 37 °C and continued to decrease at a slower pace to 0.01 mM (Fig. 3c); simultaneously, 3-HPA became detectable right after mixing (Fig. 3c) of pure acrolein with LB medium. 3-HPA concentrations reached a peak at 40 min (0.3 mM) and dropped to below 0.05 mM thereafter. Acrolein concentrations were higher when 3-HPA had been added (0.01–0.05 mM) then when reuterin had been (0.01–0.02 mM). These data clearly indicate that 3-HPA and acrolein readily interconvert at 37 °C in bacterial culture medium, resulting in the formation of free acrolein from pure 3-HPA and vice-versa the formation of 3-HPA from pure acrolein.

#### Detection of components of the reuterin system by NMR

The components of the reuterin system were analyzed simultaneously by acquisition of ^1^H, ^13^C, DQF-COSY, HSQC, and HMBC NMR spectra for a 3-HPA solution in phosphate buffer (100 mM, pH 7) at 25 °C. The three most abundant species in solution were 3-HPA, its hydrate and a cyclic dimer (Figure S3). In the dimer, the hemi-acetal proton was identified to occupy axial position on the basis of its ^3^J_HH_ coupling constants (dd, J = 9.8, 2.5 Hz). The relative concentrations of these three most abundant components were roughly 10:5:1. At least three other signals between 85 and 100 ppm were present in the ^13^C-spectrum. These and the less intense signals below 70 ppm are expected to belong to minor multimeric forms of 3-HPA and their hydrates (see also Burgé *et al*.^17^).

Supported by HSQC and COSY analysis, ^13^C-NMR signals at 199.1, 141.7, and 137.4 ppm were assigned to the fourth key component, acrolein (Figure S3a). The concentration of acrolein relative to the hydrate of 3-HPA was estimated to be less than 5% on the basis of ^13^C signal intensity (100 mM 3-HPA, relaxation delay of 30 s). Integration of the proton NMR signals recorded with pre-saturation of the water signal and within a few minutes of introducing the sample suggested a relative concentration of about 3% (Figure S3b). Thus, relying solely on ^13^C spectra, or using moderately sensitive instrumentation, small amounts of acrolein would go unnoticed, and for reliable analysis of reuterin solutions, ^1^H NMR with effective H_2_O pre-saturation is key for addressing the complexity of the system (Figure S3b).

#### Analysis of GSH-acrolein adducts

Additional evidence for the presence of acrolein in 3-HPA solutions at various pH and temperatures was obtained by MRM UPLC-ESI-MS/MS analysis of GSH-acrolein adducts being formed when the scavenger GSH is added to acrolein containing solutions 21,45,46. The MS fragmentation behavior of GSH and of *S*-(3-oxopropyl)-GSH adducts were determined in solutions of mixed GSH and acrolein that were incubated at 37 °C for 3 h. Fragmentation patterns were determined to be 308 → 179 *m/z* and 364 → 217 *m/z*, respectively (Table 2). The LOD for acrolein on the basis of its GSH conjugate was 25 nM. This level of sensitivity was sufficient to detect the conjugate in samples of reuterin, acrolein, and 3-HPA at pH 7 and at pH 4. GSH-acrolein adducts were present in acrolein, reuterin and 3-HPA samples at 37 °C and in reuterin and acrolein samples at 4 °C (Table 2). Higher temperature (37 vs. 4 °C) and pH (7 vs 4) resulted in higher relative amounts of GSH-acrolein adducts (Table 2). However, in 3-HPA samples at pH 4 stored at 4 °C, no ions corresponding to GSH-acrolein adducts could be detected confirming the absence of acrolein at these conditions (Table 2).

### Role of acrolein in PhIP transformations

To test the involvement of acrolein in the conversion of PhIP to PhIP-M1, PhIP was reacted with a reuterin solution, with pure HPA or pure acrolein under various conditions (Fig. 4). When PhIP was mixed with a solution of reuterin or acrolein at 37 °C, it was transformed to PhIP-M1 (Fig. 4e,f). When PhIP was mixed with pure 3-HPA not containing any acrolein at 4 °C (temperature at which no acrolein formed, Table 2), no PhIP-M1 was detected (Fig. 4a). We confirmed that the reaction of PhIP with acrolein proceeds at 4 °C, leading to the consumption of PhIP and formation of PhIP-M1 in an acrolein concentration-dependent manner (Fig. 4b–d). After 48 h almost complete transformation (98%) of PhIP to PhIP-M1 was observed for 100 μM PhIP and 10 mM acrolein. Over the same time period, lower extents of conversion were observed with lower acrolein concentrations: 29% for 1 mM acrolein, and 5% for 0.1 mM acrolein. Glycerol alone did not react with PhIP under any of the conditions (Figure S4).

### Bacterial inhibitory activity of reuterin, 3-HPA and acrolein solutions with and without the addition of selective acrolein scavenger compounds

Antibacterial activity is an important functionality of reuterin. To determine the role of acrolein in inhibition of bacteria of reuterin solutions, we conducted minimal inhibitory concentrations (MIC) assays using *Escherichia coli* and *Listeria innocua* as indicator strains. Reuterin, (acrolein-free) 3-HPA and acrolein were assessed in the presence and absence of acrolein scavengers GSH and *N*-acetyl-l-cysteine (NAC) on 96-well plates. In a parallel experiment changes of free unbound acrolein was monitored over time with IC-PAD. Hereby, reuterin, 3-HPA and acrolein solutions were mixed with LB medium and incubated at 37 °C in the absence of indicator strains. Acrolein was quantified at 40 min intervals and the highest acrolein concentration determined in LB media was used for the calculation of MICs of the indicator strains at test conditions.

IC-PAD investigations resulted in two findings that were important for the interpretation of the antimicrobial activity of the reuterin system components: firstly, IC-PAD revealed the formation and presence of acrolein in the non-inoculated bacterial culture media after 3-HPA and reuterin were added, and interestingly even after the addition of initially acrolein-free 3-HPA solutions (Fig. 3). Secondly, IC-PAD experiments confirmed the selective acrolein scavenging of GSH and of NAC (Fig. 2b,c): when added to reuterin, no acrolein was detectable in solution while 3-HPA concentration was not changed 1 h after addition of GSH or NAC (Fig. 2b,c).

MIC values reported for acrolein concentration in the test of reuterin, 3-HPA and acrolein with and without the addition of selective acrolein scavenger compounds are presented in Table 3. MICs of acrolein depended on whether reuterin or 3-HPA solutions, or pure acrolein were applied, and were 5–14 times higher for acrolein than for 3-HPA. Interestingly, when selective acrolein scavengers GSH or NAC were added, acrolein, reuterin and 3-HPA solutions completely lost antimicrobial activity. This result combined with the previous finding that after the addition of NAC and GSH no acrolein was detectable in solution any more, points at acrolein as the active antimicrobial compound.

## Discussion

Already a hundred years ago, Voisenet discovered the formation of acrolein when a *Bacillus* species was grown in the presence of glycerol and it was hypothesized that glycerol was first dehydrated to 3-HPA before acrolein was formed in a secondary reaction^31^,^32^. Similarly, acrolein was present in a *Lactobacillus* culture that was cultivated in the presence of glycerol in 1960^47^.

Here, a novel analytical approach was used that (1) allowed the concurrent detection of 3-HPA and acrolein in reuterin solutions and (2) was able to detect acrolein at minimum concentrations of 25 nM. NMR results matched well the results reported by Vollenweider *et al*.^16^ for 3-HPA in deuterium oxide (D_2_O) at similar concentrations. Binding of acrolein and GSH to result in *S*-(3-oxopropyl)-*N*-acetyl-l-cysteine or *S*-(3-oxopropyl)-GSH was shown in IC-PAD as well as MRM UPLC-ESI-MS/MS experiments in this study confirmed previous reports^21^,^45^,^46^. GSH and GSH-acrolein adduct fragments used for MRM UPLC-ESI-MS/MS experiments were well in agreement with fragments observed by Oberth and Jones^45^.

Using this combined approach, we investigated the conditions and rates for the conversion of 3-HPA to acrolein and established that from a pure sample of 3-HPA, acrolein forms on a timescale of hours in buffered solution as well as in bacterial culture media. As a consequence of this interconversion, the presence of acrolein and its contribution to activities attributed to 3-HPA has remained undiscovered and might have biased analytical methods employed. For example it can be suggested that 3-HPA is converted to acrolein in the widely used TrpHCl test^38^, which involves an incubation step at 37 °C; thus, apparent 3-HPA levels measured in this test represent in fact acrolein which had formed from 3-HPA.

Similar to previous findings^48^ the equilibrium position of 3-HPA and acrolein was pH independent at near-neutral pH. No change of the equilibrium position was detected between 25 and 45 °C. Low temperature slowed the interconversion, which is indicative of the exothermic character of the reaction^16^,^41^,^49^,^50^. The hydration rates of acrolein increased between pH 6 and 9 at 37 °C, confirming previous studies at similar conditions^48^,^49^. The acrolein preparation used contained 0.2% hydroquinone as a stabilizer to prevent polymerization and the reaction with water to form 3-HPA which might interfere with the determination of acrolein-3HPA interconversion^37^. However, the commercial product was diluted at least 1500 fold (v/v) during the preparation of working solutions. The amount of hydroquinone became too low to confer stabilizing activity and to interfere with our experiments^37^.

Acrolein is a highly reactive and electrophilic α,β-unsaturated aldehyde and, on the basis of its structure, is readily anticipated to be more reactive than 3-HPA with regards to nucleophile addition. Acrolein adducts of GSH and NAC formed quickly consistent with previous effective use of scavengers to prevent the induction of hepatotoxicity by acrolein^20^,^51^,^52^,^53^. Similarly, binding to SH-containing amino acids and peptides present in LB medium probably led to the rapid decline of acrolein concentrations observed during inhibition testing (Figure S2) and therefore also impacted the determination of MICs as suggested by large standard deviations.

Acrolein is proposed to cause oxidative stress in bacteria by reaction with free thiol groups^21^,^53^. Total intracellular thiol content in *E. coli* cells decreased to about 20% of initial levels after exposure to acrolein^53^, and GSH-deficient *E. coli* mutants were significantly more susceptible to acrolein when compared to a wild type strain^21^,^53^. However, intracellular GSH content alone cannot explain differences in sensitivity as there was no correlation observed between intracellular concentrations of low molecular weight thiols including GSH and the sensitivity toward reuterin of various bacterial strains^23^.

The supposition of acrolein as the antibacterial compound in reuterin solutions appears to contradict previous studies that addressed the mechanism of antibacterial activity of reuterin and identified 3-HPA as the active component^20^,^21^. GSH supplementation apparently decreased the inhibitory activity of 3-HPA toward *E. coli* 21. However, these studies lacked the analytical tools to determine that acrolein is rapidly formed from initially acrolein-free 3-HPA solutions after addition to culture medium and during incubation. In light of the new information obtained here, it can be concluded that acrolein was present under the conditions used, and that after GSH addition, an inactive GSH-acrolein conjugate was formed.

Another process previously attributed to HPA is the conversion of the HCA PhIP to its glycerol conjugate PhIP-M1. PhIP-M1 is formed from PhIP in a microbial process that requires glycerol and the production of reuterin^24^,^26^. The transformation has been observed in growing cultures of *L. reuteri* and of *E. hallii*, as well as in batch fermentations of fecal and colon microbiota^24^,^26^. Here we identified acrolein as the compound that reacts with PhIP. It can be suggested that PhIP conjugation occurs by direct reaction with acrolein via the proposed chemical mechanism illustrated in Fig. 5. Stepwise 1,4-addition of the HCA to acrolein is followed by ring closure via 1,2-addition of the 2-imino group to the intermediate aldehyde (Fig. 5). It is well established that acrolein reacts with primary and secondary amines, including lysine, histidine, and imidazole, as well as with thiols, like cysteine and glutathione^51^,^52^,^53^,^54^. Furthermore, adducts similar in structure to PhIP-M1 were identified as products of reactions of acrolein with deoxyguanosine^55^, arginine^56^, cytosine, and adenine^57^.

The observation that PhIP-M1 is formed by fecal and colon microbiotas^24^,^25^, and that a substantial proportion of 3-HPA is converted to acrolein at conditions prevailing in the human colon as observed in this study, suggests that acrolein might derive from microbial glycerol-dependent 3-HPA formation *in vivo*. In agreement, PhIP-M1 could be recovered from feces of consumers that obtained a single portion of cooked chicken meat^25^. Indeed, several gut microbes beside *L. reuteri* and *E. hallii* such as *Ruminococcus obeum, Ruminococcus gnavus, Flavonifractor prautii, Intestinimonas butyriciproducen*s, *E. coli* and *Veillonella* spp. harbor glycerol/diol dehydratases and thus have the potential to metabolize glycerol to 3-HPA which can then dehydrate to acrolein^9^.

This suggests that acrolein is constantly formed in the gut keeping in mind that gut microbiota composition is individually distinct in humans^58^. Reuterin and acrolein produced *in vivo* could on one site confer increased ecological competitiveness of the producer cell due to its antimicrobial property. On the other hand, acrolein-HCA conjugation has relevance to human toxicity as mutagenicity of PhIP, and of structurally related HCAs, requires hydroxylation of the imidazole primary amine^59^. This chemical pathway is effectively blocked by conjugation of HCAs with acrolein, which could lead to a natural detoxification step. Therefore, intestinal glycerol metabolism to 3-HPA and acrolein might establish a basis on how microbial communities may mitigate HCA or induce acrolein exposure^25^,^60^,^61^.

**Figure 1:**
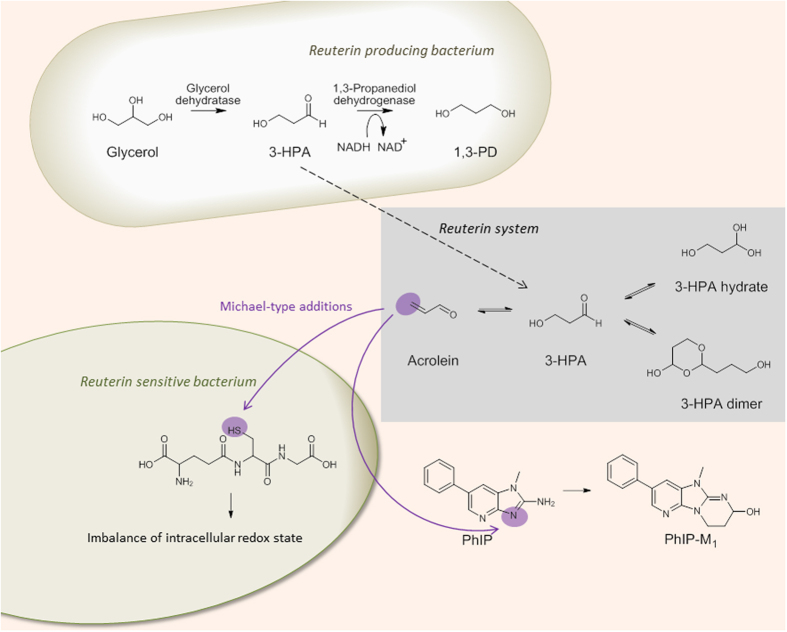
Components of the reuterin system and proposed mechanism of activity. Reuterin producing bacteria excrete 3-hydroxypropionaldehyde (3-HPA) which in solution forms reuterin. Reuterin is comprised of 3-HPA, its hydrate 1,1,3-propanetriol, the dimer 2-(2-hydroxyethyl)-4-hydroxy-1,3-dioxane and acrolein. Acrolein is proposed to be the active compound causing antibacterial activity as well as the conversion of heterocyclic amines (HCAs).

**Figure 2:**
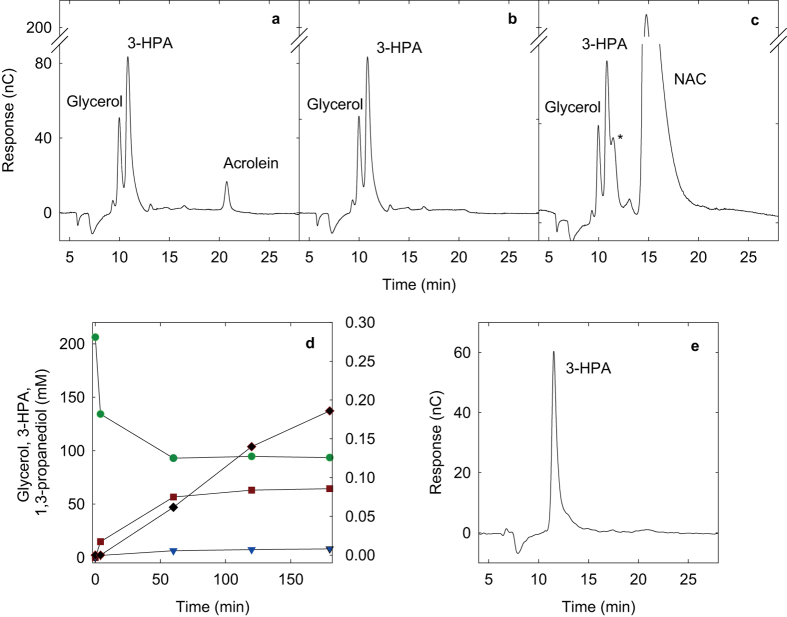
Identification and quantification of 3-HPA and acrolein using IC-PAD. (**a**) Reuterin solution containing 3-HPA, acrolein, 1,3-propanediol (1,3-PD) and residual glycerol after conversion of glycerol by *Lactobacillus reuteri* DSM 20013, (**b**) acrolein-free reuterin solution after addition of glutathione (GSH), (**c**) acrolein-free reuterin solution after addition of *N*-acetyl cysteine (NAC); peak marked with the asterisk is supposed to be representative of the NAC-acrolein adduct, (**d**) reuterin production process over a 3 h period at 25 °C, glycerol (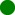), 3-HPA (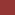), 1,3-PD (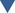), and acrolein (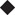), for a distinct illustration the result of a single production is shown (in total: n = 2), (**e**) 3-HPA solution after solid-phase extraction (n ≥ 2).

**Figure 3:**
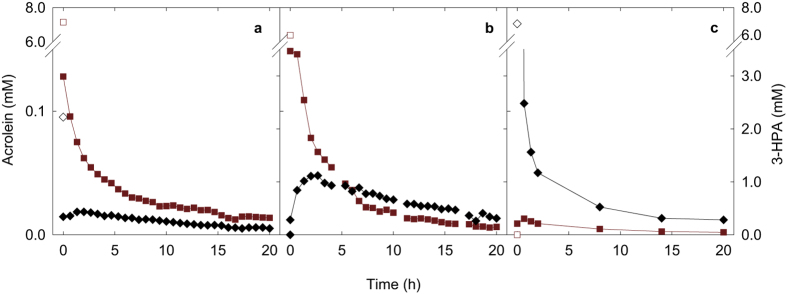
Kinetic behavior of acrolein and 3-HPA added to LB culture medium at 37 °C. Acrolein (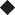) and 3-HPA concentrations (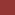) were analyzed when (**a**) a reuterin solution, (**b**) 3-HPA and (**c**) acrolein were added to LB medium (pH 6.8). Initial concentrations of acrolein and 3-HPA in aqueous solutions prior to the addition to LB medium (open symbols). To allow better readability, different scales were chosen for acrolein and 3-HPA data and in (**c**) acrolein data point 7.1 mM is not shown but is included in the figure at the end of the dashed line.

**Figure 4:**
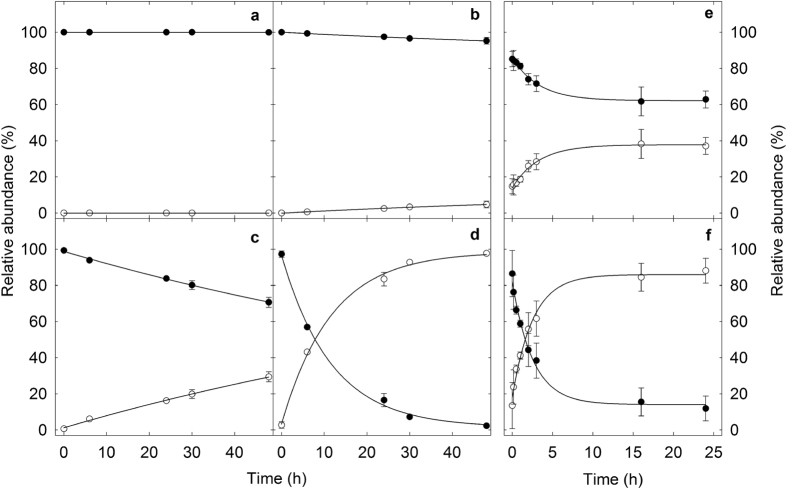
Reactivity of 3-HPA and acrolein with PhIP. Changes in relative abundance of PhIP (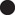) and PhIP-M1 (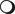) in the presence of (**a**) 10 mM 3-HPA, (**b**) 0.1 mM acrolein, (**c**) 1.0 mM acrolein, and (**d**) 10.0 mM acrolein at 4 °C and pH 4 and in the presence of (**e**) reuterin and (**f**) 10 mM acrolein at 37 °C and pH 7. Standard deviations are displayed but are too small to be visible for some data points (n = 3).

**Figure 5:**
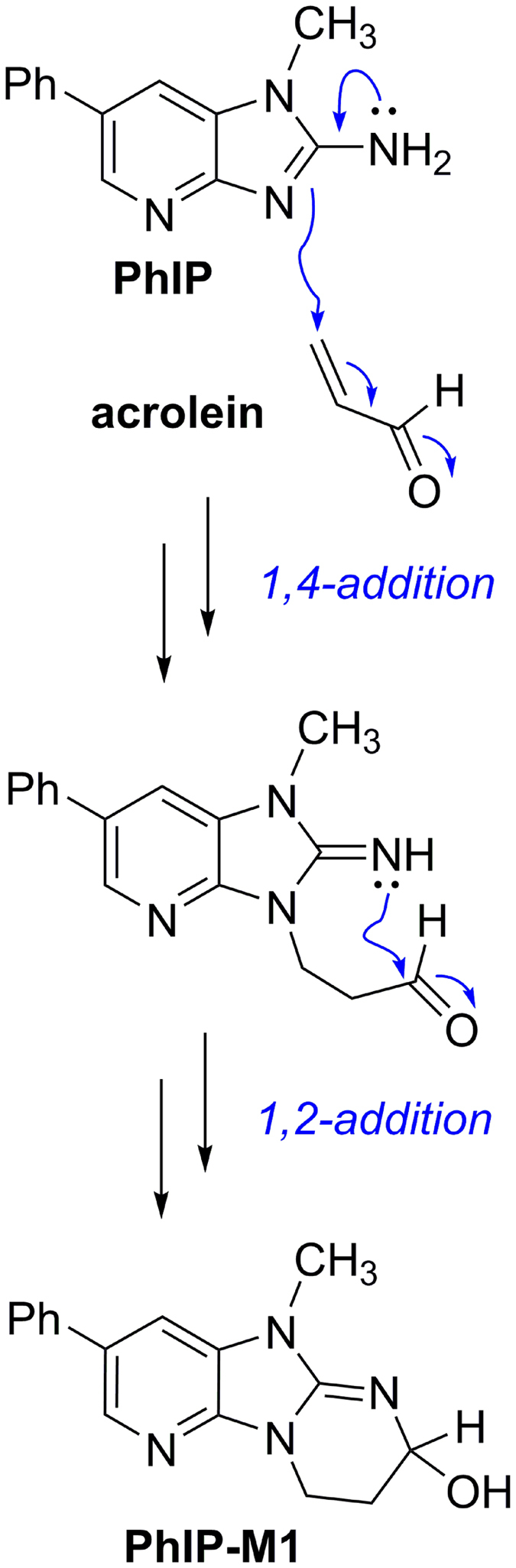
Suggested chemical mechanism for the reaction of PhIP with acrolein. 1,4-conjugate addition of acrolein and PhIP to result in PhIP-M1.

In conclusion, integrating data resulting from multiple chemical analyses enabled detailed kinetic characterization of the 3-HPA/acrolein interconversion and confirmed the presence and rapid formation of acrolein from 3-HPA at physiological conditions. Furthermore, the molecular basis of activities related to antimicrobial activity and HCA conjugation were elucidated (Fig. 1). As formation of the HCA conjugate was previously observed *in vivo,* our results imply that acrolein is formed in the human gut with potential implications on the detoxification on HCAs, as well as on enhancing competitiveness of the producer strain. As a consequence we propose to re-define the term reuterin to describe a multi-compounds system containing 3-HPA, its hydrate, the dimer and also acrolein.

## Methods

### Hazard information

Acrolein is acutely toxic (oral, dermal, inhalation), forms flammable gases, is corrosive and hazardous to the aquatic environment. PhIP is a possible human carcinogen (IARC group 2B^62^). Appropriate personal protective equipment was used.

### Chemicals

2-Amino-1-methyl-phenylimidazo[4,5-b]pyridine (PhIP) was from Toronto Research Chemicals (Toronto, ON, Canada); D_2_O was from Armar AG (Döttingen, Switzerland); acetonitrile was from Merck KGaA (Darmstadt, Germany) and formic acid was from Fisher Scientific (Geel, Belgium). Acrolein (>99%, product number: 89116) was purchased from Sigma-Aldrich GmbH (Buchs, Switzerland) and contained 0.2% hydroquinone as stabilizer. All other chemicals were also purchased from Sigma-Aldrich Chemie GmbHunless stated otherwise.

### Reuterin production and 3-HPA purification

Reuterin was produced (*n* = 2) from glycerol using *Lactobacillus reuteri* DSM 20016^T^ (DSMZ, Deutsche Sammlung von Mikroorganismen und Zellkulturen, Braunschweig, Germany) and 3-HPA was isolated from resulting reuterin solutions as described previously^16^.

In brief, *L. reuteri* was grown in MRS medium (Biolife, Milan, Italy) supplemented with 20 mM glycerol (Sigma-Aldrich, Buchs, Switzerland) for about 14 h at 37 °C before cells were harvested and washed twice in 100 mM potassium phosphate buffer (pH 7). Cells were re-suspended in sterile 200 mM glycerol solution before conversion of glycerol to reuterin was conducted at 25 °C for 3 h. Reuterin-containing supernatant was recovered by centrifugation, followed by filtration (0.2 μm) and lyophilisation.

Pre-purification to eliminate the orange-red coloured impurities was conducted in a Büchner funnel filled with silica gel 60; fractions were generated employing with acetone-ethyl acetate (2:1, Fisher Scientific, Loughborough, UK; Sigma-Aldrich). Fractions containing 3-HPA were pooled before all solvents were evaporated *in vacuo* until approximately 10 mL remained that were loaded onto a silica gel 60 column. Fractions were eluted using acetone-ethyl acetate (2:1) and 3-HPA content was monitored with IC-PAD as indicated below. Solutions containing only 3-HPA were pooled, all solvents were evaporated *in vacuo* to dryness and finally traces of acetone and ethyl acetate were evaporated under reduced pressure. The purity of the remaining substance was verified with NMR spectroscopy, whereby special attention was paid to confirm the initial absence of acrolein (as described below, data not shown).

### Indicator strains and culture conditions

Non-pathogenic strains *Escherichia coli* DSM 5698 and *Listeria innocua* DSM 20649^T^, closely related to common food pathogens, were used as indicator strains in inhibitory tests and grown under aerobic conditions in lysogeny broth (pH 6.8, LB medium, Becton, Dickinson and Co, Sparks, MD, USA) at 37 °C and 30 °C, respectively. Stock cultures were maintained at −80 °C in 30% glycerol.

### Ion-exclusion chromatography with pulsed-amperometric detection (IC-PAD)

IC-PAD analysis was performed on a Thermo Scientific (Reinach, Switzerland) ICS-5000+ system equipped with a quaternary gradient pump, a thermostated autosampler and an electrochemical detector with a cell containing a Ag/AgCl reference electrode and a disposable thin-film platinum working electrode tempered at 25 °C. Analytes were separated with a Thermo Scientific IonPac ICE-AS1 4 × 250 mm ion-exclusion column with a guard column, operated at 30 °C. The solvent system was isocratic 0.1 M methanesulfonic acid at 0.2 mL min^−1^ for 36 min. The injection volume was 10 μL. A knitted reaction coil was placed between column and detector to minimize dissolved oxygen from the sample. Purified water sparged with helium (18 MΩ * cm resistivity) was used to prepare the eluent. Electrochemical data were obtained after modification and optimization of the triple-potential waveform consisting of regeneration/detection, oxidation and reduction potentials.

External calibration was performed using dilutions of freshly prepared reference standards of acrolein, 3-HPA, glycerol and 1,3-propanediol. All dilution steps were performed on ice to minimize evaporation, samples were placed in the cooled autosampler in airtight liquid chromatography (LC) vials right before the analysis. Water was used as solvent. Detection and quantification limits were determined based on signal-to-noise ratios (>3:1 and >10:1, respectively) according to the ICH guideline^62^. Control standards were repeatedly measured between samples to detect and control changes in electrode signal. System control, data acquisition and processing were performed using Chromeleon 7 Chromatography Data Software.

### Analysis of 3-HPA/acrolein interconversion

IC-PAD was used to follow the kinetics for the 3-HPA/acrolein interconversion at different temperatures (4, 25, 37, and 45 °C). Solutions of 3-HPA and acrolein were prepared in phosphate buffer (20 mM, pH 6, pH 7, and pH 8) and borate buffer (20 mM, pH 9) to reach initial concentrations of 4 mM and 1 mM, respectively. 3-HPA and acrolein content was analyzed over the course of 24 h (for 37 and 45 °C) and 48 h (for 4 and 25 °C). Acrolein is an extremely volatile compound, to minimize acrolein evaporation airtight LC vials were used for analysis. The absence of acrolein smell during the experiment verified the suitability of this approach. The experiments were performed at least in triplicate.

**Figure 6:**
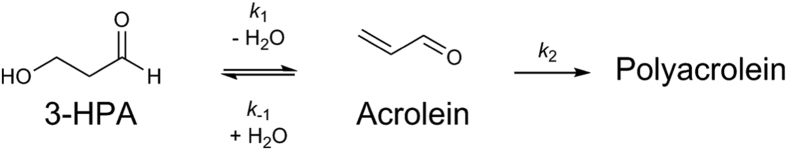
Model used for fitting the kinetic data.

Experimental data for concentrations of acrolein and 3-HPA were fit to a pseudo-first-order model. In each experiment a distinct deviation from 100% mass balance was observed, and the missing mass was attributed to the formation of polyacrolein. Polymerization of acrolein has been observed in previous studies 41,42,43,44. Rate constants (*k*) and equilibrium constants (K) were determined from these fits. The kinetic model used is shown in Fig. 6; methods used for fitting the kinetic data are described in the supplementary methods section.

Additionally, the fate of reuterin, 3-HPA and acrolein was characterized under conditions mimicking those used during microbial inhibition testing, i.e. addition to LB medium (pH = 6.8) at 37 °C. The amount of 3-HPA and acrolein in LB medium to which one of the three test solutions had been added, was directly analyzed in undiluted samples at 40 min intervals with IC-PAD over 20 h. In addition, similar experiments were conducted using 10-fold dilutions and shortened experiment times (i.e. 120 min for reuterin, 200 min for 3-HPA and 80 min for acrolein). The experiment was performed in triplicate.

### NMR spectroscopy

Samples were prepared by adding 5% D_2_O to a freshly prepared solution of 3-HPA (100 mM) in phosphate buffer (100 mM, pH 7). ^13^C NMR spectra were recorded at 150 MHz on a Bruker AVIII 600 MHz spectrometer (Fällanden, Switzerland) equipped with a CPDCH He-cooled cryoprobe. Spectral width was 248 ppm, the transmitter was set to 110 ppm. 768 scans were accumulated with an acquisition time of 0.87 s per scan. ^1^H-decoupling was on during acquisition only and the relaxation delay between scans was set to 30 s in order to allow reliable quantification.

All other spectra (^1^H, DQF-COSY, HSQC, HMBC) were recorded on a Bruker AVIII HD 600 MHz spectrometer equipped with a Prodigy N_2_(l)-cooled triple resonance cryoprobe. The ^1^H spectrum was recorded with presaturation of the water signal using a 1D-NOESY sequence (mixing time of 10 ms). Acquisition time was 5.5 s for 128 k data points. 32 scans were accumulated with an interscan delay of 7.5 s (presaturation time). The transmitter was set to 4.7 ppm (water signal) with a spectral width of 20 ppm. DQF-COSY: 8192 × 512 data points, 8 scans per increment. Spectral width was 10.5 ppm, the transmitter was set to 4.7 ppm. Water suppression was achieved using excitation sculpting. HSQC: 2048 × 512 data points, 8 scans per increment. Spectral width (^1^H/^13^C) was 14 and 160 ppm, the transmitter was set to 4.7 and 80 ppm, respectively. Water suppression was achieved using the WATERGATE element. HMBC: 2048 × 512 data points, 16 scans per increment. Spectral width (^1^H/^13^C) was 12.2 and 240 ppm, the transmitter was set to 4.7 and 109 ppm, respectively. Water suppression was achieved by presaturation. Acquisition of all experiments was performed within one day at 25 °C.

### Detection of acrolein as its GSH adduct by MRM UPLC-ESI-MS/MS

The formation of GSH-acrolein adducts was monitored with multiple reaction monitoring ultra-performance liquid chromatography electrospray ionization tandem mass spectrometry (MRM UPLC-ESI-MS/MS) on a Waters nanoAcquity Ultra Performance LC (Waters AG, Baden-Dättwil, Switzerland) coupled with a Thermo LTQ Velos ion trap mass spectrometer fitted with an ESI source.

GSH-acrolein was separated from GSH on a Phenomenex Synergi Fusion RP 80A (500 μm i.d. × 150 mm, 4 μm particle size) kept at 25 °C. The injection volume was 1 μl. The flow rate was set to 5 μL min^−1^ and compounds were eluted with 20 mM ammonium formate in water (eluent A) and acetonitrile (eluent B). The gradient program was as follows: 0% (10 min), 0–90% B (1 min), 90% (2 min), 0% (1 min), followed by re-equilibration.

Positive ion spectra were recorded in the range of *m/z* 100−800 applying the following parameters: capillary temperature, 220 °C; spray voltage, 3.0 kV; collision energy, 19 eV (GSH) and 16 eV (GSH-adducts) and sheath gas pressure, 5 AU. Transition reaction parameters were optimized using standards of GSH and acrolein in a 1:1 ratio (1 mM each). Thermo Xcalibur software was used for system control, data acquisition and processing.

Evidence for the presence of acrolein was the detection of GSH-acrolein adducts by MRM mass spectroscopy. GSH was added to solutions of 3-HPA (21 mM 3-HPA, 1 mM GSH), reuterin (21 mM 3-HPA, 1 mM acrolein, 1 mM GSH) or acrolein (1 mM acrolein, 1 mM GSH) in 20 mM phosphate buffer at pH 4 or pH 7. All samples were prepared freshly and kept on ice during sample preparation. Thereafter, samples were incubated for 3 h at 4 °C or 37 °C prior to UPLC-ESI-MS/MS analysis. The detection limit was determined based on signal-to-noise ratio (>3:1) according to the ICH guideline^63^. The experiment was performed in triplicate.

### Analysis of PhIP and PhIP-M1 with UPLC-ESI-MS/MS

PhIP and PhIP-M1 were analyzed using the same LC-MS instrumental set-up as described in the previous section. Compounds were separated on a Waters Acquity BEH130 C18 column (300 μm i.d. × 150 mm, 1.7 μm particle size) kept at 40 °C. The injection volume was 1 μL. The flow rate was 5 μL min^−1^, and compounds were eluted with 0.1% formic acid and 10% acetonitrile in water (v/v, eluent A),50) and 0.1% formic acid in acetonitrile (v/v, eluent B). The gradient program was as follows: 0–3 min: 0% B, 3–13 min: 0–95% B (10 min), 13–18 min: 95% B, 18–19 min: 95–0% B, followed by re-equilibration.

Positive ion spectra were recorded in the range of *m/z* 100−600 applying the following parameters: capillary temperature, 250 °C; spray voltage, 3.5 kV; collision energy, 35 V and sheath gas pressure, 20 AU. The MS ionization parameters were optimized by tuning with a 1 μM PhIP solution in 50% methanol in 0.1% formic acid in water. Thermo Xcalibur software was used for system control, data acquisition and processing.

PhIP transformation activities by 3-HPA or acrolein were evaluated in phosphate buffer (20 mM) at pH 4 and 4 °C. 3-HPA was investigated at a final concentration of 10 mM, acrolein concentrations at 0.1, 1.0 and 10 mM and PhIP at 100 μM. Concentrations of PhIP and PhIP-M1 were determined immediately after mixing with 3-HPA or acrolein and after 6, 24, 30, and 48 h. Additionally, to determine the influence of elevated temperature, PhIP transformation activities by reuterin solutions and acrolein (10 mM) were evaluated at 37 °C. Hereby, concentrations of PhIP and PhIP-M1 were analyzed immediately after mixing and after 10 min, 0.5, 1, 2, 3, 16, and 24 h at pH 7. Glycerol (10 mM) was employed as control. The experiment was performed in triplicate.

### Bacterial inhibitory activity of reuterin, 3-HPA and acrolein solutions with and without the addition of selective acrolein scavenger compounds

Inhibitory activities of reuterin solutions, 3-HPA and acrolein toward *E. coli* and *L. innocua* with and without the addition of selective scavengers were determined with critical-dilution assays on 96-well plates.

Hereby, first, to determine the amount of unbound acrolein present during inhibition testing (i.e. at a given ratio of acrolein to media compounds, temperature, etc.) reuterin, 3-HPA and acrolein solutions were mixed with LB medium and incubated at 37 °C for 80, 200 and 160 min, respectively, in the absence of indicator strains. Acrolein concentrations were determined every 40 min using IC-PAD and the highest acrolein concentrations determined were considered to be present in the first well and were used for the calculation of those acrolein present in other wells on 96-well plates: acrolein concentrations tested ranged from 1 to 735 μM in reuterin solutions, from 2 to 645 μM in 3-HPA solutions and from 3 to 1130 μM in acrolein solutions.

Two-fold serial dilutions of 3-HPA, reuterin and acrolein were inoculated with overnight cultures of the indicator strains to a cell count of approximately 10^7^ CFU mL^−1^ and incubated overnight at 30 °C (*L. innocua*) or 37 °C (*E. coli*). Bacterial growth was detected by measuring the optical density at 600 nm after 20 to 22 hour of incubation. A sigmoidal (four parameter logistic) equation was used to fit the data (Sigma Plot version 12); the inflection point of the resulting curve represents the MIC50,acrolein value which were defined as the acrolein concentration that reduced final optical density of the indicator strains to 50% compared to final optical density of the indicator strains in LB broth.

NAC and GSH were added in about 20-fold concentration of acrolein to selectively scavenge this compound and therefore to enable the investigation of acrolein-free 3-HPA. Samples were checked for the presence of acrolein before and 1 h after the addition of scavengers at 37 °C. NAC and GSH alone did not inhibit the growth of indicator strains. Experiments were done at least in triplicate.

## Additional Information

**How to cite this article**: Engels, C. *et al*. Acrolein contributes strongly to antimicrobial and heterocyclic amine transformation activities of reuterin. *Sci. Rep.* **6**, 36246; doi: 10.1038/srep36246 (2016).

**Publisher’s note**: Springer Nature remains neutral with regard to jurisdictional claims in published maps and institutional affiliations.

## Supplementary Material

Supplementary Information

## Figures and Tables

**Table 1:** Temperature and pH-dependency of the 3-HPA/acrolein interconversion.

Temperature (°C)	pH	*k*_1_ (10^−6^ s^−^1)	*k*_−1_ (10^−6^ s^−^1)	*k*_2_ (10^−6^ s^−^1)	K = k_1_/k_−1_
4	7	≤0.07^a^	0.45 ± 0.05^a^	2.0 ± 0.2^a^	≤0.15
25	7	0.84	7.32	8.07	0.11
37	6	3.19	20.37	12.8	0.16
37	7	5.39	33.08	16.6	0.16
37	8	4.86	30.14	14.7	0.16
37	9	13.31	83.11	27.3	0.16
45	7	7.42	51.98	8.76	0.14

Rate constants *k* and equilibrium constants *K* were determined at various conditions. Samples at pH 4 and 4 °C were also investigated, but as no conversion was detectable within a 48 h period it was not possible to calculate a definitive value for *k* or *K* (n ≥ 3).

^a^These parameters were determined based on the formation of 3-HPA in the acrolein sample.

**Table 2:** Detection of acrolein-GSH adducts.

Conditions	Proportion of acrolein-GSH adducts detected in solutions [in %]			
		Reuterin	3-HPA	Acrolein
pH 4	4 °C	13 ± 4	<1^1^	34 ± 16
pH 4	37 °C	43 ± 19	9 ± 7	72 ± 15
pH 7	37 °C	99 ± 2	92 ± 8	100 ± 0

LC-MRM-MS detection of ions representing acrolein-GSH adducts calculated as proportions of acrolein-GSH adducts relative to all GSH detected in percent. The formation of acrolein-GSH adducts after scavenger addition verified the presence of acrolein or its formation from 3-HPA during incubation (n = 3).

^1^0.2 ± 0.1 mM.

**Table 3:** Antibacterial activity of acrolein-containing solutions.

Strains	MIC_50_ (μM)								
	Reuterin			3-HPA^1^			Acrolein		
	None	GSH	NAC	None	GSH	NAC	None	GSH	NAC
*L. innocua*	42.6 ± 26.1	—2	—2	10.1 ± 0.3	—2	—2	137.0 ± 46.6	—2	—2
*E. coli*	34.6 ± 8.5	—2	—2	13.5 ± 1.7	—2	—2	61.9 ± 27.0	—2	—2

Minimum inhibitory concentrations (MIC_50_s) of acrolein present in reuterin or 3-HPA solution, or of pure acrolein toward two indicator strains in the absence (None) and presence of scavenger compounds glutathione (GSH) and *N*-acetyl cysteine (NAC). Acrolein was quantified in culture medium after reuterin, 3-HPA and acrolein solutions were supplied. Shown are mean MIC_50_ of at least three independent experiments.

^1^IC-PAD investigations showed rapid acrolein formation after addition of 3-HPA solutions to LB medium.

^2^No inhibition of bacterial growth.
