# Supplementary material for: Acrolein contributes strongly to antimicrobial and heterocyclic amine transformation activities of reuterin
Source: Sci Rep. 2016 Nov 7;6:36246. doi: 10.1038/srep36246 (PMC5098142; doi:10.1038/srep36246)
Supplement: Supplementary Information [file srep36246-s1.doc]

# Supplementary material for:

**Acrolein contributes strongly to antimicrobial and heterocyclic amine transformation activities of reuterin**

Christina Engels^1^, Clarissa Schwab^1^, Jianbo Zhang^2^, Marc J. A. Stevens^1^, Corinne Bieri^1^, Marc-Olivier Ebert^3^, Kristopher McNeill^4^, Shana J. Sturla^2^, Christophe Lacroix^1#^

^#^ corresponding author,

^1^ Laboratory of Food Biotechnology, Institute of Food, Nutrition and Health, Department of Health Sciences and Technology, ETH Zurich, Zurich, Switzerland

^2^ Laboratory of Food Nutrition and Toxicology, Institute of Food, Nutrition and Health, Department of Health Sciences and Technology, ETH Zurich, Zurich, Switzerland

^3^ Laboratory of Organic Chemistry, Department of Chemistry and Applied Sciences, ETH Zurich, Zurich, Switzerland

^4^ Laboratory of Environmental Chemistry, Institute of Biogeochemistry and Pollutant Dynamics, Department of Environmental Systems Science, ETH Zurich, Zurich, Switzerland

Running title: Acrolein confers activities attributed to reuterin

# Supplementary methods

### Methods for fitting the kinetic data

The kinetic model used is shown in the following:


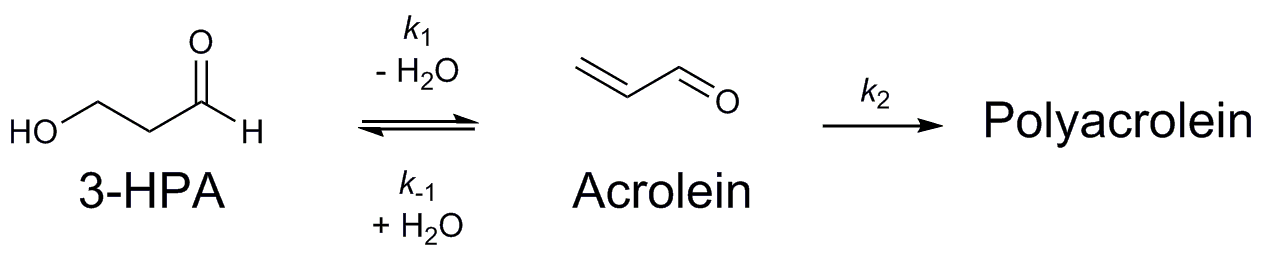


The equilibrium constant K was defined as K = *k* _1_ /*k* _-1_. When starting from 3-HPA (and [acrolein]_0_ = 0), we used the analytical solution shown in Figure S1. When starting from acrolein (and [3-HPA]_0_ = 0), numerical kinetic modeling was used to fit the data. A global kinetic fit approach was employed, whereby *k* _1_, *k*_- 1_ and *k*_2_ were shared between data sets.

**Figure S1 | Analytical solution**. This model was used to fit data when starting from 3-HPA (and [acrolein]_0_ = 0; taken from Espenson (1981[^1^](#_ENREF_3)) with slight modifications.

**
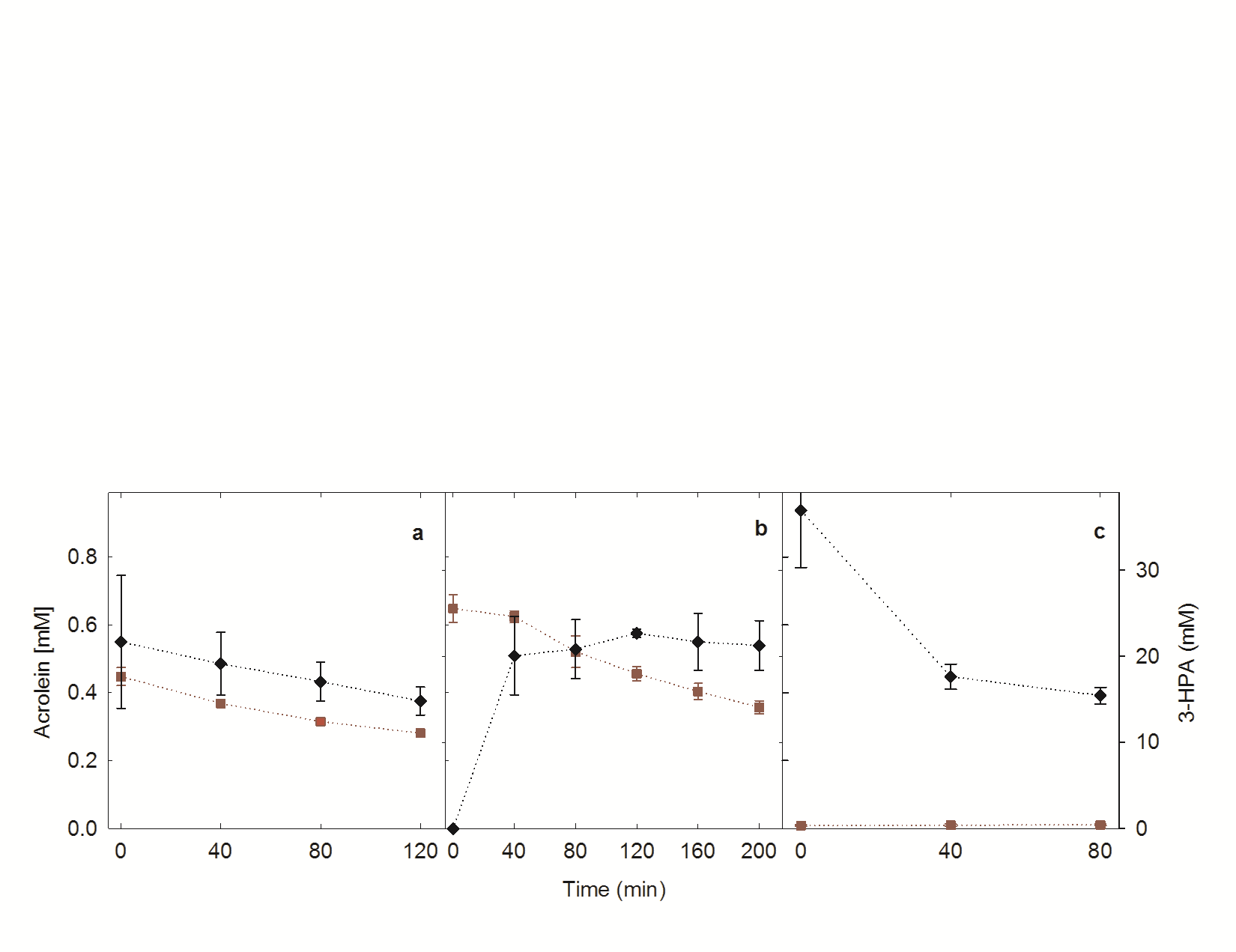
**

**Figure S2 | Kinetic behavior of acrolein and 3-HPA added to LB culture medium at 37 °C.** Acrolein (◆) and 3-HPA concentrations (◼) were analyzed when (**a**) a reuterin solution, (**b**) 3-HPA and (**c**) acrolein were added to LB medium (pH 6.8); samples were analyzed after 10-fold dilution (n = 3).


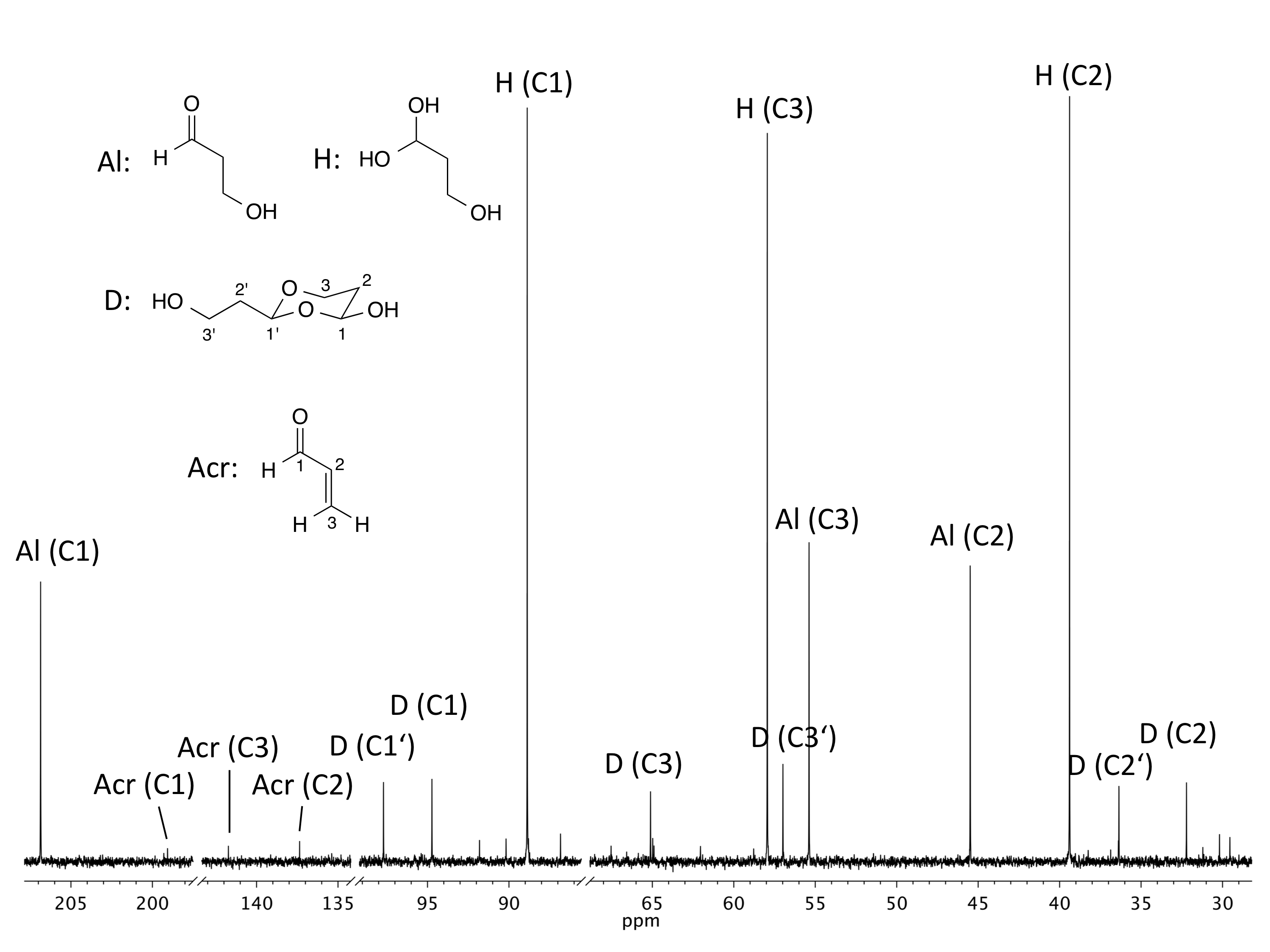


**a**


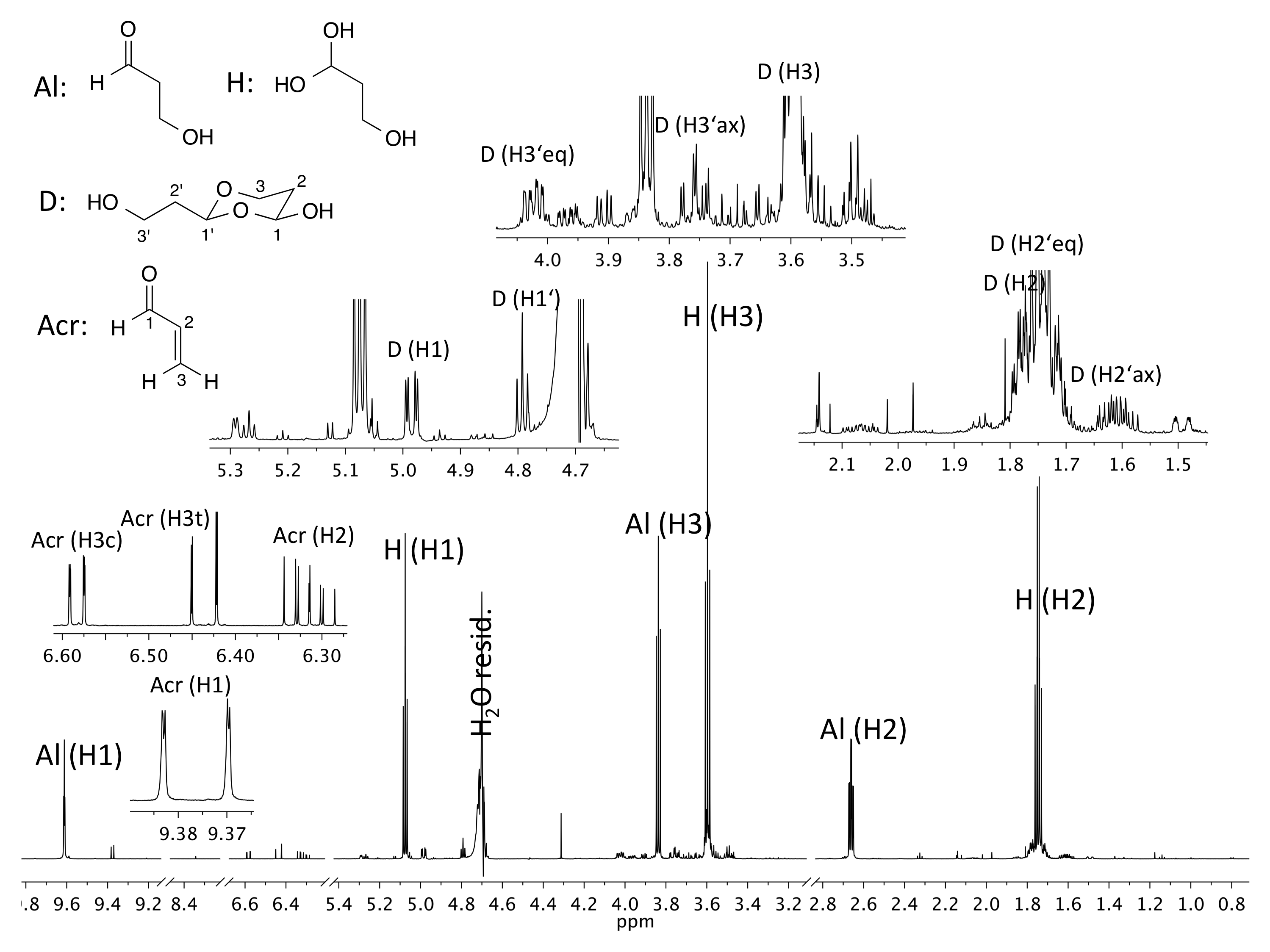


**b**

**Figure S3 | ^13^C NMR (a) and ^1^H NMR (b) spectrum of a reuterin sample.** Peaks shown represent 3-HPA (Al), its hydrate 1,1,3-propanetriol (H) and the dimer 2-(2-hydroxyethyl)-4-hydroxy-1,3-dioxane (D) as well as acrolein (Acr).

**
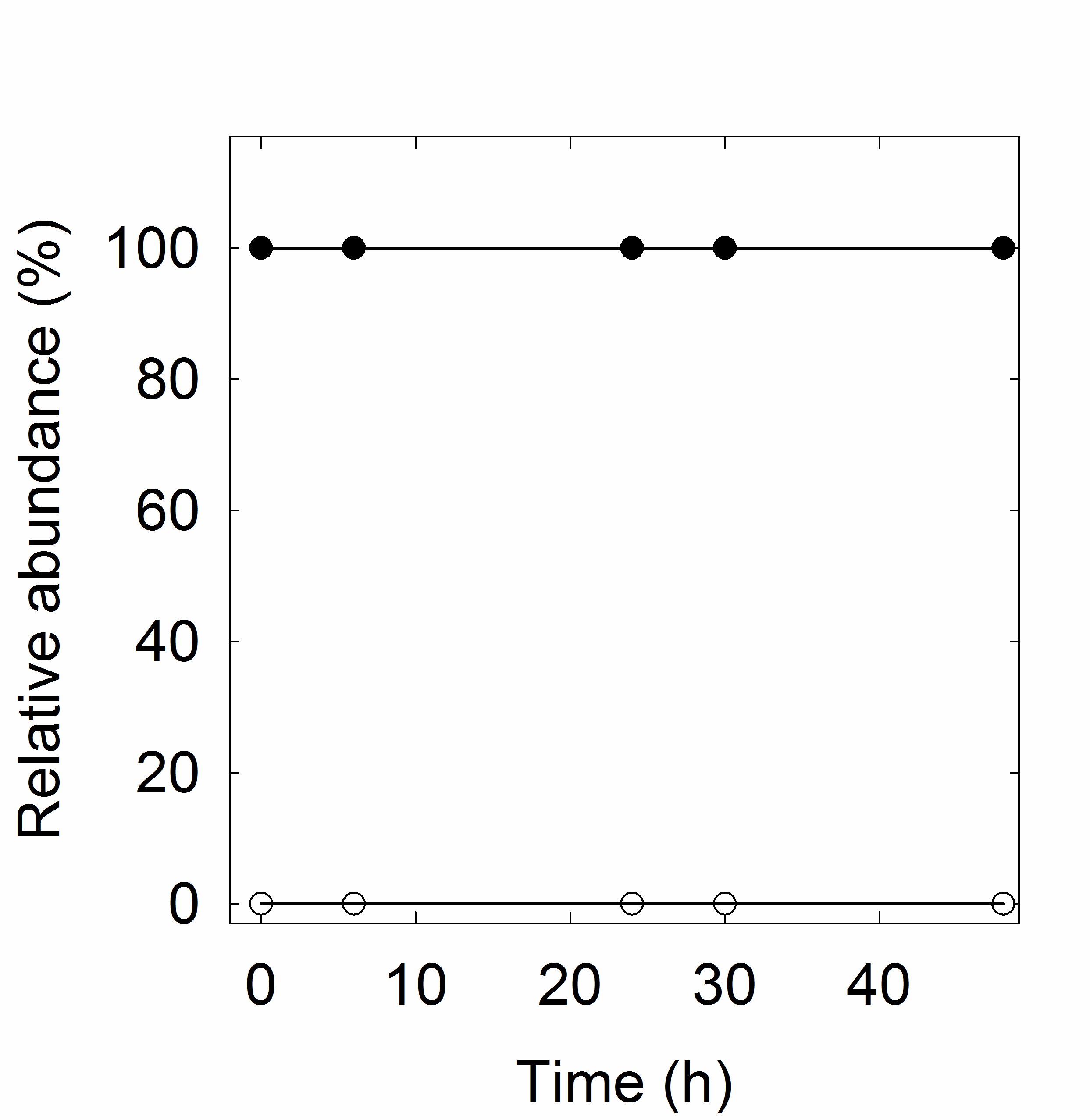
**

**Figure S4 | Reactivity of glycerol with PhIP.** Changes in relative abundance of PhIP (filled circles) and PhIP-M1 (open circles) in the presence of 10 mM glycerol at 4 °C and pH 4. Standard deviations are displayed but are too small to be visible for some data points (n = 3).

## References

1 Espenson, J. *Chemical Kinetics and Reaction Mechanisms*. 2nd edn, p. 77 (McGraw-Hill Education, 1981).
